# DRSeg: a weakly supervised framework for breast ultrasound image segmentation

**DOI:** 10.1038/s41598-026-49728-0

**Published:** 2026-04-28

**Authors:** Meng Xu, Bin Hu, Yingfeng Wang, Patrick Koo, Kuan Huang

**Affiliations:** 1 Department of Computer Science and Technology, Kean University, Union, 07083 USA; 2 Department of Computer Science and Engineering, University of Tennessee at Chattanooga, Chattanooga, 37403 USA; 3 University of Tennessee Health Science Center College of Medicine-Chattanooga, Chattanooga, 37403 USA; 4 Division of Pulmonology and Critical Care, Erlanger Health, Chattanooga, 37403 USA

**Keywords:** Breast ultrasound, Weakly supervised segmentation, Image selection, Pseudo-label generation, Computational biology and bioinformatics, Engineering, Health care, Mathematics and computing, Medical research

## Abstract

Automatic segmentation of breast tumors in ultrasound images can assist physicians in making accurate and effective decisions. However, fully supervised learning methods typically require large amounts of pixel-level annotations, which are often difficult to obtain. Weakly supervised segmentation using image-level labels offers a promising alternative, but generating high-quality pseudo-segmentation labels remains a major challenge. In this study, we propose DRSeg, a weakly supervised segmentation framework for breast ultrasound (BUS) images that explicitly addresses the variability of pseudo-label quality. DRSeg augments a standard CAM-based pipeline with four components: (1) Class Activation Map (CAM) generation and refinement; (2) a Dual-ROI selection algorithm that identifies images with stable CAM localizations for reliable pseudo-label generation; (3) pseudo-label generation using the Segment Anything Model; and (4) training a segmentation model with the Mean Teacher strategy, leveraging both pseudo-labeled and non-pseudo-labeled images. Extensive experiments on two public BUS datasets, BUSI and BLU, demonstrate the effectiveness of the proposed DRSeg framework and its individual components. Using Swin Transformer V2 as the backbone, DRSeg achieves an IoU of 59.35% and an F1 score of 69.27% on BUSI. Using ResNet-50 as the backbone, DRSeg achieves an IoU of 51.79% and an F1 score of 60.00% on BLU, outperforming six existing weakly supervised methods. Overall, DRSeg reduces reliance on pixel-wise annotations while achieving competitive segmentation performance across two public BUS datasets and two backbones.

## Introduction

Breast cancer is a major global health concern, particularly among women. In the United States, it is projected to have the highest number of new cases among all cancer types in 2024 and is the second leading cause of cancer-related deaths among the top ten cancers affecting women^1^. The incidence of breast cancer has been on the rise since the mid-2000s, primarily attributed to changes in dietary habits, lifestyle choices, and delays or absences in childbirth. However, the mortality rate has decreased by 44% since 1989, primarily due to advancements in early detection methods^2^. Breast ultrasound (BUS) imaging plays a vital role in early breast cancer detection due to its portability, radiation-free nature, affordability, and suitability for women with dense breast tissue^3,4^. However, BUS images are inherently challenging to interpret because of their characteristics such as low contrast, speckle noise, and acoustic shadows. As a result, accurate diagnosis typically requires the expertise of experienced radiologists. Automatic computer-aided diagnosis systems can enhance the interpretation of BUS images, provide second opinions, and support radiologists in making more precise diagnoses, particularly for accurately segmenting tumor edges in BUS images^5^.

Despite their clinical value, medical image segmentation remains challenging due to boundary ambiguity and complex visual structures^6,7^. To address these challenges, BUS image segmentation has been extensively studied using both traditional machine learning and deep learning techniques. Earlier approaches employed traditional methods such as support vector machines^8^, K-means, and fuzzy C-means^9^. More recently, deep learning models have become dominant, including convolutional neural network-based techniques like U-Net^10^ and Transformer-based models such as TransUNet^11^. Fully supervised image segmentation methods have been successfully applied to BUS image segmentation and achieved promising results. However, these methods require pixel-level annotations for training, which are time-consuming and labor-intensive to generate. As a result, acquiring large-scale BUS image datasets with pixel-level annotations becomes a significant challenge, limiting the research progress of automated BUS image segmentation.

To address the annotation challenge, semi-supervised image segmentation^12^ and weakly supervised image segmentation methods^13^ have been developed. Weakly supervised image segmentation typically eliminates the need for pixel-level annotations by leveraging image-level or text-level annotations, which are easier to obtain and have therefore gained significant attention. These methods have primarily evolved into two main categories: multi-instance learning^14,15^ and CAM-based approaches^16^. Among these, CAM-based approaches are particularly attractive due to their ability to significantly reduce the time, cost, and memory requirements associated with image segmentation. This has led to the development of various CAM techniques, such as CAM^16^, Grad-CAM^17^, and Grad-CAM++^18^.

Despite their advantages, most existing CAM-based weakly supervised segmentation methods have been developed and validated primarily on natural images. Only limited studies have investigated weakly supervised BUS image segmentation using image-level annotations^19^, leaving a notable gap between existing methodology and clinical ultrasound applications. More broadly, recent studies have explored attention-based designs in medical image segmentation, highlighting the importance of reliable localization under limited supervision^20,21^.

CAM-based weakly supervised segmentation approaches^22–24^ generally follow a common multi-stage procedure: 1) train a classification model with image-level labels; 2) generate CAMs using the trained model; 3) refine the CAMs, 4) create pseudo pixel-level labels from the refined CAMs; and 5) train a segmentation model with these pseudo labels. As a result, the performance of the final segmentation model heavily depends on the quality and reliability of the generated pseudo labels, which are directly determined by the quality of the underlying CAMs.

In practice, however, the reliability of CAM-derived pseudo labels varies substantially across images, especially in noisy modalities such as breast ultrasound. This observation motivates the key questions addressed in this work: whether the stability of CAMs across different threshold choices can reliably indicate pseudo-label quality, and whether selectively training on images exhibiting such stability can improve weakly supervised segmentation performance.

**Fig. 1:**
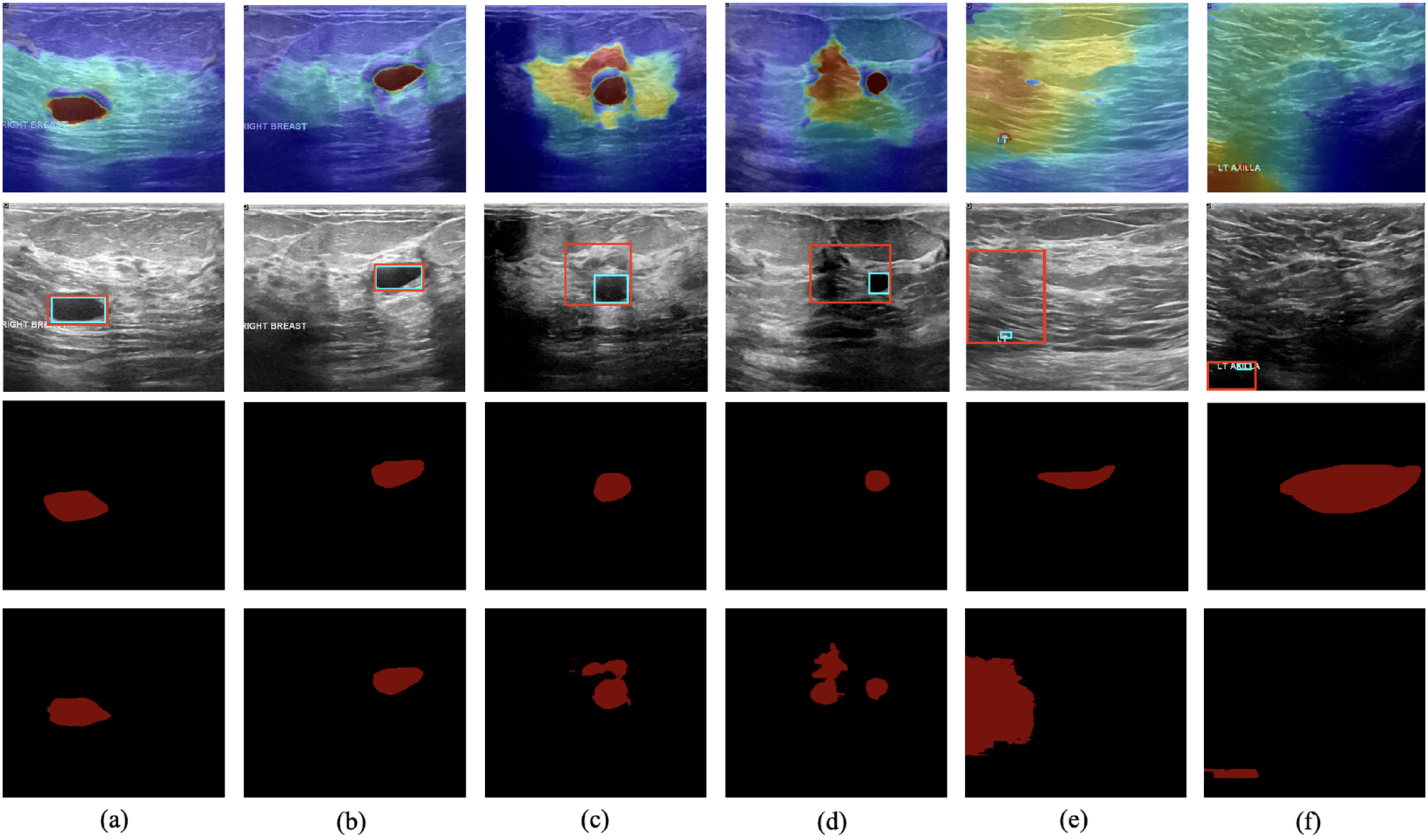
Examples of BUS images from dataset BUSI^25^ with their corresponding Grad-CAMs^17^ and pseudo segmentation labels of varying quality. The first row displays the Grad-CAMs. The second row shows the bounding boxes generated using two different thresholds applied to the Grad-CAMs: red boxes correspond to a lower threshold, while blue boxes correspond to a higher threshold. The third row presents the ground truth segmentation labels, and the fourth row presents the pseudo segmentation labels generated from the corresponding Grad-CAMs.

To improve CAM quality and thereby improve the resulting pseudo-pixel-level labels, various refinement techniques have been proposed, such as online data augmentation^26^, mix-up methods^27,28^, Inter-pixel Relation Network (IRNet)^29^, and conditional random fields^30^. Nevertheless, our empirical observations indicate that generating consistently high-quality CAMs across all images in a BUS dataset remains challenging. As illustrated in Fig. 1, the reliability of CAM-derived pseudo labels varies substantially across images. Based on these observations, we categorize BUS images into three groups:

- For images such as Figs. 1(a) and 1(b), high-quality CAMs are generated, and the resulting pseudo labels remain stable and of high quality, regardless of the threshold values used.
- For images such as Figs. 1(c) and 1(d), the quality of the CAMs is moderate, making the pseudo-labels highly sensitive to threshold selection. High-quality pseudo-labels can only be obtained with appropriately chosen threshold values.
- For images such as Figs. 1(e) and 1(f), the CAMs are of poor quality, and reliable pseudo-labels cannot be generated, regardless of the threshold values applied.

Including images from the latter two categories (Figs. 1 c–f) during pseudo-label generation introduces noise, which can degrade the training of the final segmentation model. This observation motivates the need to selectively identify images that are suitable for pseudo-label generation, rather than treating all samples equally.

To address this challenge, we propose Dual-ROI Segmentation (DRSeg), a weakly supervised segmentation framework specifically designed for breast ultrasound imaging. Rather than introducing a new segmentation architecture, DRSeg introduces a selection-driven training strategy that improves pseudo-label reliability prior to segmentation model training. Specifically, the DRSeg framework consists of four key components: (1) CAM generation using Grad-CAM^17^ with SwinV2 Tiny^31^ and CAM refinement using IRNet^29^; (2) an innovative Dual-ROI selection algorithm to identify the most suitable images for pseudo-label generation; (3) pseudo label generation using the Segment Anything Model^32^; and (4) training a segmentation model, DeepLabV3^33^, with the Mean Teacher strategy^34^, utilizing both pseudo-labeled and non-pseudo-labeled images in the training set. Our major contributions are as follows:

- Proposing DRSeg, a weakly supervised framework for BUS image segmentation that combines CAM-based pseudo labeling with a Mean Teacher strategy to effectively utilize both labeled and non-pseudo-labeled data.
- Proposing a novel Dual-ROI selection algorithm that identifies images suitable for reliable CAM-based pseudo-label generation and can be readily integrated into existing CAM-based weakly supervised segmentation pipelines.
- Benchmarking four backbone models, including VGG-16^35^, ResNet-50^36^, SwinV2 Tiny^31^, and VmambaV2 Tiny^37^, for CAM generation. Our results show that SwinV2-Tiny produces the most effective pseudo labels for training the DeepLabV3 segmentation model.
- Evaluating DRSeg on two publicly available BUS image datasets, BLU^38^ and BUSI^25^. Experimental results demonstrate the effectiveness and generalizability of DRSeg while reducing the need for costly pixel-level annotations.

The proposed DRSeg framework is at a proof-of-concept stage, evaluated retrospectively on publicly available breast ultrasound datasets, and is currently in pre-clinical development. DRSeg is intended as a pre-clinical training framework to facilitate the development of breast ultrasound tumor segmentation models when only image-level labels are available, with the goal of reducing reliance on costly pixel-level annotations. By lowering the annotation burden, DRSeg supports pre-clinical model development and retrospective analysis in settings where expert annotations are limited.

## Related work

### Weakly supervised image segmentation

Weakly supervised image segmentation aims to reduce the reliance on pixel-level annotations by leveraging weaker forms of supervision, such as image-level labels^23^, text descriptions^39,40^, or bounding boxes^41^. This approach is particularly attractive in medical imaging, where generating pixel-level labels is highly time-consuming and requires expertise from experienced physicians. Recent surveys and studies have systematically explored weak supervision strategies across natural and medical image domains, highlighting their potential and limitations^42,43^. Among various weak supervision forms, image-level labels are the easiest to obtain but challenging to implement effectively for accurate localization.

Existing weakly supervised segmentation methods based on image-level supervision generally fall into two categories: CAM-based and Multiple Instance Learning (MIL)-based approaches. MIL-based methods enable semantic segmentation by treating an image as a bag and its patches or pixels as instances within the MIL framework. Many MIL-based approaches have been proposed for medical image segmentation. For example, Lerousseau *et al.*^44^ proposed a weakly supervised framework for whole-slide image segmentation, leveraging typical clinical annotations to generate patch-level predictions. Qian *et al.*^45^ developed a MIL framework for whole-slide image segmentation by integrating the Swin Transformer with the MIL concept. Similarly, Lai *et al.*^46^ proposed a Transformer-based multiple superpixel-instance learning approach for lung disease segmentation in CT images, incorporating superpixels into the MIL framework to enhance weakly supervised segmentation performance. Overall, MIL methods treat patches or pixels as individual instances, making them effective for images with distinct structural features, such as whole-slide pathology or CT images. However, in BUS images, the low contrast and subtle texture variations make it difficult to define meaningful and separable instances, limiting the effectiveness of MIL-based approaches in this domain.

CAM-based methods instead rely on classification networks to generate coarse localization maps that highlight discriminative regions associated with image-level labels. Early works such as CAM^16^, Grad-CAM^17^, and its extension Grad-CAM++^18^ have formed the foundation for this research field. Subsequent efforts have focused on improving CAM quality. For example, techniques such as expanding highlighted regions by erasing the most distinctive features have been introduced in works like ACoL^47^ and EIL^48^. In addition, multi-branch learning with Activation Modulation and Recalibration (AMR) scheme^23^ and post-processing with affinity learning^29^ and CRFs^30^ have also been explored. While these approaches improve spatial coverage and boundary consistency, they generally assume that all training images can eventually yield usable pseudo labels after refinement.

In the context of breast ultrasound imaging, only limited work has explored weakly supervised segmentation using image-level annotations^49^. These studies primarily focus on CAM refinement or auxiliary anatomical constraints, but still rely on the assumption that refined CAMs across the dataset are sufficiently reliable for pseudo-label generation. In practice, however, CAM quality varies substantially across BUS images, even when advanced refinement techniques are applied. This observation motivates a complementary perspective: instead of refining all CAMs, selectively identifying images that can generate reliable CAMs may be a more effective strategy for weakly supervised segmentation.

### Image selection and sample reliability in weak supervision

Image selection is an important step in many machine learning workflows, such as data curation, active learning, and semi-supervised or weakly supervised learning, where selecting informative or reliable samples can significantly improve training efficiency. An effective selection strategy ensures that the training dataset contains representative and high-quality samples, and therefore improves the model performance. Various approaches have been proposed for image selection, such as heuristic-based, uncertainty-based, and diversity-based methods. For instance, Gao *et al.*^50^ introduced a consistency-based semi-supervised active learning framework, which prioritizes images exhibiting the highest prediction inconsistency across augmentations. Variational Adversarial Active Learning^51^ uses a variational autoencoder and an adversarial network to identify representative and informative images. Additionally, other selection strategies have been explored, including information-theoretic approaches^52^, ensemble-based methods^53^, uncertainty heuristics^54^, and learning-based techniques like loss prediction^55^.

Despite their effectiveness, these general-purpose selection methods are not designed to address the pseudo-label reliability problem in CAM-based weakly supervised segmentation. In particular, they do not explicitly consider the stability of spatial localization under varying CAM thresholds, which directly affects pseudo-label quality. Recent efforts in prompt selection for foundation models such as SAM focus on learning optimal prompts^22^, but they typically assume reliable prompts are already available and do not address the upstream issue of selecting images suitable for pseudo-label generation.

In contrast to prior work that focuses on improving CAM generation or refining pseudo segmentation labels through post-processing, this study shifts the attention to pre-segmentation image selection. Rather than uniformly refining CAMs for all images, we explicitly identify images whose CAMs exhibit stable spatial localization across different threshold choices. Such stability provides an indicator of pseudo-label reliability and enables more effective downstream segmentation training. By framing image selection as a reliability filtering step specific to CAM-based weak supervision, DRSeg complements existing CAM refinement and teacher–student training strategies.

## Methods

### Overview

DRSeg is a training framework rather than a new segmentation architecture. It operates by augmenting a standard CAM-based weakly supervised pipeline with an explicit image selection stage prior to pseudo-label generation and segmentation training. The core idea is to improve pseudo-label reliability by identifying images whose CAM localizations are spatially stable, rather than attempting to refine CAMs for all images uniformly.

**Fig. 2:**
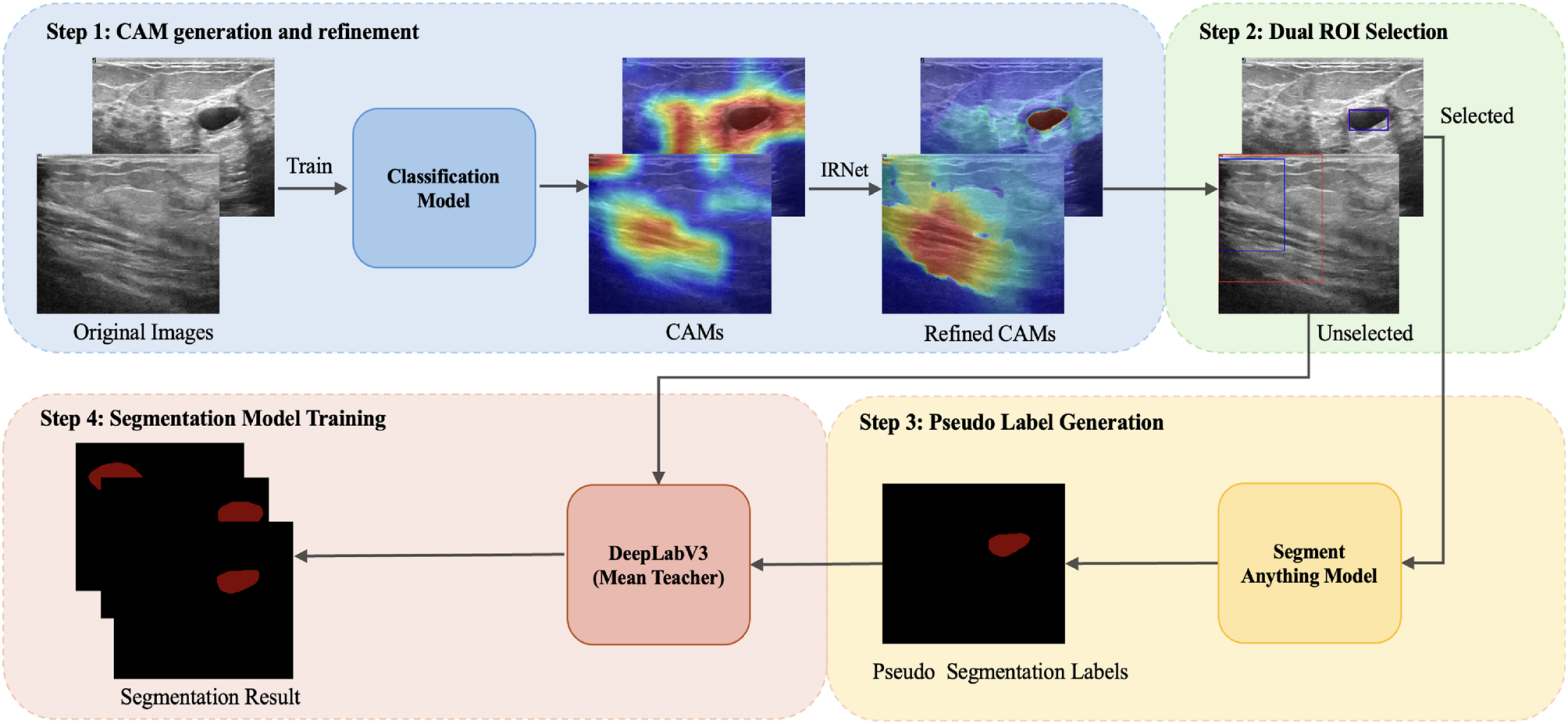
The overview of the proposed method.

As illustrated in Fig. 2, the proposed Dual-ROI Segmentation (DRSeg) framework consists of four major steps: (1) CAM generation and refinement, (2) image selection using the proposed Dual-ROI selection algorithm, (3) pseudo-label generation, and (4) training a segmentation model employing the Mean Teacher strategy to utilize both pseudo-labeled and non-pseudo-labeled images in the training set.

Let the complete dataset be defined as $D = \{ D_{\text {train}}, D_{\text {test}} \}$, where $D_{\text {train}}$ is used for training and $D_{\text {test}}$ for evaluation. The following sections provide details on each major step.

### CAM generation and refinement

The CAM generation and refinement stage aims to produce high-quality visual explanations for tumor localization, enabling the generation of reliable pseudo-segmentation labels in later stages. Specifically, we first train a classification model on $D_{\text {train}}$ and generate CAMs using the Grad-CAM method^17^. We then refine these CAMs using IRNet^29^. The choice of classification model backbone is described in the Results section.

First, a classification model $f_{\text {cls}}$ is trained on the training set $D_{\text {train}} = \{(x_i, y_i) \mid i = 1, 2, \dots , N\}$, where $x_i$ represents the input image, $y_i \in \{0, 1\}$ is the class label ($0$ for benign and $1$ for malignant tumors), and $N$ is the total number of training samples. The model is optimized using the cross-entropy loss function:

1 $$\begin{aligned} L_{\text {cls}} = -\frac{1}{N} \sum _{i=1}^N \left[ y_i \log \hat{y}_i + (1 - y_i) \log (1 - \hat{y}_i) \right] , \end{aligned}$$

where $\hat{y}_i = f_{\text {cls}}(x_i)$ is the model’s predicted probability for the given input $x_i$. Once the model converges, it is used to compute CAMs for tumor localization.

After training the classification model, we generate the CAM for the predicted class using the Grad-CAM approach^17^, where the predicted class is defined as:

2 $$\begin{aligned} \hat{c} = \arg \max _{c \in \{0, 1\}} \hat{y}_c \end{aligned}$$

where $\hat{y}_c$ denotes the model’s predicted probability for class $c$. The predicted class $\hat{c}$ is used as the target class for CAM generation, ensuring that the resulting CAM directly corresponds to the model’s prediction and highlights the regions most relevant to its confidence in classifying the tumor as benign or malignant.

We follow the standard Grad-CAM formulation as introduced in prior work^17^, and briefly restate it here for completeness. Given the feature maps *A* from a specific layer, the Grad-CAM importance weights $\alpha _k^{\hat{c}}$ for the predicted class $\hat{c}$ are computed as:

3 $$\begin{aligned} \alpha _k^{\hat{c}} = \frac{1}{Z} \sum _{i} \sum _{j} \frac{\partial \hat{y}_{\hat{c}}}{\partial A_{ij}^k}, \end{aligned}$$

where $Z = H \times W$ is the spatial size of the feature map, and $A_{ij}^k$ represents the activation at spatial location $(i, j)$ in the $k$-th feature map. Next, the CAM for the predicted class $\hat{c}$ is computed by:

4 $$\begin{aligned} \text {CAM}_{\hat{c}} = \text {ReLU}\left( \sum _k \alpha _k^{\hat{c}} A^k\right) , \end{aligned}$$

where the ReLU operation retains only positive contributions, highlighting the regions most relevant for the classification decision. The feature maps *A* are typically extracted from the final convolutional layer before the classification head, ensuring they contain high-level semantic information essential for meaningful CAM generation.

After generating the initial CAMs, denoted as $\textrm{CAM}_{init}$, IRNet^29^ is applied to refine them through three main stages: (1) generating inter-pixel relationship (IR) labels, (2) training IRNet using the derived affinity labels, and (3) refining the initial CAMs with the trained IRNet. To train IRNet, $\textrm{CAM}_{init}$ are processed to create IR labels through thresholding and conditional random fields. These IR labels classify pixel pairs into three categories: background positive affinity, foreground positive affinity, and negative affinity. IRNet is then trained with these affinity labels to predict both semantic affinities and displacement fields, optimizing a combined loss that includes both the affinity loss and displacement loss. After training, the refined CAMs, denoted as $\textrm{CAM}_{ref}$, are obtained by applying the trained IRNet to $\textrm{CAM}_{init}$.

In this stage, IRNet is employed to refine the initial CAMs by learning semantic affinities and displacement fields, addressing common issues of incomplete object coverage and poor boundary localization in raw CAMs. It is especially beneficial for noisy and low-contrast BUS images, as modeling local pixel relationships helps smooth noise and sharpen tumor boundaries. This refinement step produces pseudo labels that are more representative and spatially precise, providing a stronger foundation for subsequent image selection and pseudo-label generation. The output of this stage consists of refined CAMs for all images in the training set $D_{\text {train}}$.

### Dual-ROI selection

To ensure high-quality pseudo labels for training an effective segmentation model, we design a Dual-ROI selection algorithm that identifies images whose CAM localizations are spatially stable across different threshold choices, thereby selecting samples most suitable for pseudo-label generation. Intuitively, reliable CAMs exhibit consistent lesion localization under threshold perturbations, whereas large spatial divergence indicates unstable attention and unreliable pseudo masks. This stability-based criterion provides a simple and model-independent way to assess pseudo-label reliability without introducing additional supervision or architectural complexity.

The Dual-ROI algorithm is based on the regions of interest (ROIs) derived from the refined CAM of each image in $D_{\text {train}}$. First, a refined CAM is normalized to the range [0, 1]:

5 $$\begin{aligned} CAM_{\text {norm}} = \frac{CAM_{ref} - \min (CAM_{ref})}{\max (CAM_{ref}) - \min (CAM_{ref})} \end{aligned}$$

Second, each normalized CAM is binarized using two individual thresholds *thres*1 and *thres*2, where $thres1> thres2$ and both values are within the range [0,1].

6 $$\begin{aligned} & binaryImg1 = {\left\{ \begin{array}{ll} 1 & \text {if } CAM_{\text {norm}} \ge thres1 \\ 0 & \text {otherwise} \end{array}\right. } \end{aligned}$$

7 $$\begin{aligned} & binaryImg2 = {\left\{ \begin{array}{ll} 1 & \text {if } CAM_{\text {norm}} \ge thres2 \\ 0 & \text {otherwise} \end{array}\right. } \end{aligned}$$

Third, we extract an ROI from each binary image. For *binaryImg*1, we identify the minimal rectangular bounding box that fully encloses the region with a value of 1 (the highlighted area). This extracted ROI, denoted as *ROI*1, is defined by four values: $x_{min}$, $y_{min}$, $x_{max}$, and $y_{max}$, which represent the coordinates of the top-left and bottom-right corners, respectively. Similarly, we perform this extraction for *binaryImg*2 to get another ROI, denoted as *ROI*2.

Last, we calculate the Intersection of Union (IoU) between two ROIs to identify the most suitable images for pseudo label generation. For each image in the training set $D_{\text {train}}$, the IoU is calculated using the following formula:

8 $$\begin{aligned} IoU = \frac{ROI1 \cap ROI2}{ROI1 \cup ROI2} \end{aligned}$$

Images with an IoU value greater than a predefined threshold *thres*3 are selected for pseudo label generation. The subset of selected images can be defined as:

9 $$\begin{aligned} D_{pseudo} = \{ \text {image} \in D_{train} \mid \text {IoU}(\text {image})> thres3 \} \end{aligned}$$

This Dual-ROI selection algorithm is motivated by the observation that reliable CAM-based localization tends to produce spatially consistent ROIs when different thresholds are applied. As illustrated in Fig. 1, a smaller *ROI*1 extracted using a higher threshold *thres*1 is marked in red, while a larger *ROI*2 extracted using a lower threshold *thres*2 is marked in blue. When CAM localization is stable, these two ROIs exhibit a high degree of spatial overlap, as shown in columns (a) and (b). In contrast, images with low overlap between *ROI*1 and *ROI*2, as illustrated in columns (c)–(f), indicate unstable CAM localization and are therefore less suitable for reliable pseudo-label generation.

Building on this observation, the Dual-ROI selection algorithm quantifies localization stability using the IoU between *ROI*1 and *ROI*2. Images with high IoU values are selected for pseudo-label generation, while images with low IoU values are excluded from this stage. The thresholds *thres*1 and *thres*2 are chosen to represent a high-confidence core region and a more inclusive candidate region, respectively, while *thres*3 controls the strictness of selection. In practice, these parameters can be tuned using a small validation set to balance pseudo-label quality and subset size. In this study, fixed values are used across experiments to ensure reproducibility.

This stability-based selection criterion provides a simple and model-independent mechanism for improving pseudo-label reliability without introducing additional supervision or architectural complexity. The output of this stage is a selected subset of the training data, denoted as $D_{pseudo} \subset D_{train}$, which contains images with sufficiently stable ROI localization.

### Pseudo label generation

In this stage, images in the selected subset $D_{pseudo}$ are used for pseudo segmentation label generation using the Segment Anything Model (SAM)^32^. In this work, SAM is used strictly as an inference-time pseudo-label generator and is not fine-tuned on breast ultrasound data. We employ the publicly released SAM model with box prompts derived directly from the selected ROIs. No additional prompt refinement, non-maximum suppression, or post-processing steps such as conditional random fields or morphological operations are applied. The same SAM configuration is used consistently across all experiments to ensure reproducibility.

SAM is adopted for its strong zero-shot segmentation capability, which allows it to produce object masks when guided by reliable spatial prompts. However, directly applying SAM to raw BUS images without guidance often yields poor segmentation results due to low contrast, speckle noise, and ambiguous tumor boundaries. By constraining SAM with ROI-based prompts derived from stable CAM localizations, pseudo-label generation remains lightweight and reproducible, while avoiding the introduction of additional trainable components or supervision.

While SAM is used in this work, the proposed framework is not tied to a specific pseudo-label generator, and SAM can be readily replaced by other segmentation models capable of producing object masks from spatial prompts. To isolate the effect of the proposed Dual-ROI image selection strategy, SAM is used as a representative pseudo-label generator in subsequent experiments to maintain consistency.

In this work, the larger ROI (*ROI*2) is used as the prompt for SAM. Compared with the smaller ROI (*ROI*1), *ROI*2 captures both the high-confidence tumor core and its surrounding contextual region, which helps SAM generate more complete and reliable tumor masks. For each image in $D_{pseudo}$, the pseudo segmentation label is a binary mask where 0 represents the background and 1 represents the tumor, as shown in the last row of Fig. 1. As shown in columns (a) and (b), the pseudo labels generated from the images selected by the proposed Dual-ROI selection algorithm closely align with the ground truths shown in the third row. This alignment ensures effective training of the segmentation model in the subsequent stage.

The output of this stage is a set of binary pseudo segmentation masks for all images in $D_{pseudo}$, which are subsequently used as supervision for training the segmentation model.

### Segmentation model training

The training set $D_{\text {train}}$ consists of two subsets: $D_{\text {pseudo}}$, which includes images selected by the Dual-ROI algorithm for pseudo label generation, and $D_{\text {unselected}}$, which includes images not selected for pseudo-segmentation-label generation and their image-level labels remain available. In the last stage, we employ the Mean Teacher strategy to train a segmentation model using images from both $D_{\text {pseudo}}$ and $D_{\text {unselected}}$.

Specifically, the Mean Teacher strategy involves a student network and a teacher network, both implemented with identical DeepLabV3 architectures. First, images from $D_{\text {pseudo}}$ are fed into the student network, and the output is used to compute the cross-entropy loss as follows:

10 $$\begin{aligned} \mathscr {L}_{CE} = -( y \log (\hat{y}) + (1 - y) \log (1 - \hat{y}) ) \end{aligned}$$

where *y* represents the pseudo segmentation label (0 for background, 1 for tumor) and $\hat{y}$ represents the predicted probability for the tumor class.

Meanwhile, images in both $D_{\text {pseudo}}$ and $D_{\text {unselected}}$ are processed by the student network, while their noised versions are fed into the teacher network. The noise is generated by adding Gaussian noise with a standard deviation of 0.1, clipped within the range [−0.2, 0.2], to the input images. The outputs from the student network are then compared to those from the teacher network to compute a Mean Squared Error (MSE) loss:

11 $$\begin{aligned} \mathscr {L}_{MSE} = \mathscr {L}_{MSE}(s_p, t_p) + \mathscr {L}_{MSE}(s_u, t_u) \end{aligned}$$

where $s_p$ and $s_u$ represent the outputs of the student network from from $D_{\text {pseudo}}$ and $D_{\text {unselected}}$ respectively, and $t_p$ and $t_u$ are the corresponding teacher outputs. This MSE loss encourages the student network to produce predictions consistent with the teacher network.

During training, the student network’s parameters are updated by gradient descent, while the teacher network’s parameters are updated to the Exponential Moving Average (EMA) of the student network’s weights. The total loss is defined as:

12 $$\begin{aligned} \mathscr {L}_{total}=\mathscr {L}_{CE} + \alpha \mathscr {L}_{MSE} \end{aligned}$$

where $\alpha$ is a weight that balances the consistency loss $\mathscr {L}_{MSE}$ relative to the CE loss. The value of $\alpha$ is selected based on an adaptive scaling strategy, gradually increasing over training epochs. Specifically, $\alpha$ follows a linear ramp-up schedule from 0 to 0.001 over the training epochs, consistent with standard Mean Teacher implementations. This approach ensures that the model focuses on supervised learning with pseudo labels in the early stages while gradually increasing the influence of consistency regularization as training progresses.

Overall, the Mean Teacher strategy effectively leverages both the pseudo-labeled and non-pseudo-labeled images in the training set by combining supervised learning with consistency regularization. This approach helps the model learn more robust representations under weak supervision while mitigating noise in the pseudo labels, making it well suited for our weakly supervised BUS segmentation framework.

## Experiments and results

This section presents a comprehensive experimental evaluation of the proposed DRSeg framework. We first describe the datasets, data splits, evaluation metrics, and implementation details to ensure reproducibility. We then analyze key design choices, including the selection of the classification backbone for CAM generation, the Dual-ROI threshold selection strategy, and a comparison of different pseudo-label generation methods under a unified experimental setting. Next, we conduct ablation studies to quantify the contribution of individual components and examine segmentation performance under varying subset sizes selected for pseudo-label generation. We further report quantitative comparisons with existing weakly supervised segmentation methods and evaluate cross-dataset generalization. Finally, qualitative visualizations are provided to illustrate the segmentation behavior of all compared methods.

### Datasets

In this research, we utilize two public breast ultrasound datasets, BLU^38^, and BUSI^25^. The BUSI dataset contains 780 BUS images, including 210 of malignant tumors, 437 of benign tumors, and 133 of normal images. The BLU dataset contains 256 images, including 154 images of benign tumors, 98 images of malignant tumors, and 4 normal images. Since this study focuses on tumor segmentation, only images containing tumors are utilized, and normal images are excluded from this study. For each dataset, We randomly select 80% of the malignant tumor images and 80% of the benign tumor images to construct the training set $D_{\text {train}}$. The remaining images form the testing set $D_{\text {test}}$. The BUSI dataset is used for experiments on backbone model selection, optimal threshold selection for the Dual-ROI algorithm, comparison of pseudo-label generation method, the ablation study, and threshold selection for the pseudo-label subset size. Both datasets are used to compare the segmentation performance of the proposed method against peer methods.

### Evaluation metrics

We evaluate performance at both the classification and segmentation levels. For classification, we report commonly used metrics including Accuracy (ACC), Precision (PRE), True Positive Rate (TPR), False Positive Rate (FPR), F1 Score (F1), and Area Under the Curve (AUC). These metrics are used to assess the classification backbone models employed for CAM generation.

For segmentation, performance is evaluated using Intersection over Union (IoU) and F1 Score, computed at the image level and averaged over the testing set. These metrics are used consistently across all segmentation experiments reported in this work, including analyses of CAM quality through downstream segmentation performance, Dual-ROI threshold selection, ablation studies, subset size sensitivity, comparisons with existing weakly supervised segmentation methods, and cross-dataset generalization.

### Implementation details

All models are trained using PyTorch 2.8.0 on a system running Ubuntu 20.04, powered by an AMD EPYC 7513 2.60 GHz CPU and 8 NVIDIA GeForce RTX 3090 GPUs, each with 24GB of memory. All input images are resized to $224 \times 224$.

For classification models used for CAM generation, the Adam optimizer is used with an initial learning rate of 1e-4 and a weight decay of 1e-4. The learning rate is decayed by a factor of 0.1 every 20 epochs, and classification models are trained for 40 epochs. For segmentation models, including both fully supervised and Mean Teacher–based training, the same optimizer settings and learning rate schedule are used, with training conducted for 50 epochs. A batch size of 16 is applied consistently across all experiments.

When adopting the Mean Teacher strategy, an exponential moving average (EMA) decay factor of 0.999 is used for the teacher model. The consistency loss weight ($\alpha$ in Eq. 12) between the mean squared error (MSE) loss and the cross-entropy loss is gradually increased from 0 to 0.001 over the course of training using a linear ramp-up schedule. For CAM refinement, the default configuration provided in the original IRNet implementation is used without modification. Pseudo-label generation follows the procedure described in Section Pseudo Label Generation.

For the Dual-ROI selection algorithm, the thresholds, *thres*1 and *thres*2 are set to 0.9 and 0.7, respectively, to represent a high-confidence core region and a more inclusive candidate region. They are selected based on preliminary empirical analysis and fixed across all experiments. The threshold *thres*3, which controls the strictness of image selection, is chosen to balance pseudo-label quality and subset size and may vary across backbone models and datasets. In this study, *thres*3 is set to 0.1 when using SwinV2 as the classification backbone and 0.5 when using ResNet-50, as discussed in Comparing Segmentation Performance with Varying Subset Sizes. Fixed threshold values are used consistently across experiments to ensure reproducibility.

For experiments in which randomness may affect performance, all models are trained and evaluated across five random seeds, and results are reported as mean ± standard deviation. This approach is used consistently across ablation studies, segmentation performance assessment of DRSeg under varying subset sizes selected for pseudo-label generation, and comparisons with peer methods.

For all peer methods included in the comparative evaluation, we used their official implementations with author-recommended hyperparameters, with minor tuning of key hyperparameters to ensure stable convergence. This approach is applied consistently to ensure fair and reproducible comparisons with the proposed DRSeg framework.

### Backbone selection for the classification model

This section discusses the backbone selection for CAM generation, as presented in the CAM Generation and Refinement section. To determine the optimal backbone for CAM generation in our study, we compare the performance of four commonly used classification models: VGG-16^35^, ResNet-50^36^, Swin Transformer V2 tiny (SwinV2)^31^, and Vmamba V2 tiny (VmambaV2)^37^. Each model is independently trained on the training set $D_{\text {train}}$ and subsequently used to generate CAMs for all images within $D_{\text {train}}$. These CAMs are then processed by the IRNet^29^ for refinement. The refined CAMs are binarized at a fixed threshold of 0.7 to produce a single ROI for each image. This threshold is applied consistently across backbone models to isolate the effect of backbone choice and is further analyzed in Section Comparing Segmentation Performance with Varying Subset Sizes. Subsequently, these ROIs serve as prompts for the SAM model^32^ to generate pseudo labels, which are subsequently used to train a DeepLabV3^56^ model for breast tumor segmentation. It is important to note that, in this backbone selection process, all images in $D_{\text {train}}$ are used without applying the Dual-ROI selection algorithm, and only one ROI is extracted per image. Furthermore, the Mean Teacher training strategy is not used in this phase. These design choices allow the performance of the backbone models to be evaluated without the influence of additional selection or training strategies.

To evaluate the quality of CAM generation by these backbone models, we compare both their classification performance and the final segmentation performance produced by DeepLab, which utilizes pseudo labels generated from these models. The performance of each model is evaluated on the testing set $D_{\text {test}}$ (Fig. 2).

**Table 1:** Classification Performance for Different Backbones.

	ACC$\uparrow$	PRE$\uparrow$	TPR$\uparrow$	FPR$\downarrow$	F1$\uparrow$	AUC$\uparrow$
SwinV2	0.9000	0.9149	0.9451	0.2051	0.9297	0.9377
VmambaV2	0.9231	0.9355	**0.9560**	0.1538	0.9457	0.9348
ResNet-50	**0.9462**	**0.9773**	0.9451	**0.0513**	**0.9609**	**0.9549**
VGG-16	0.8692	0.9302	0.8791	0.1538	0.9040	0.9166

**Fig. 3:**
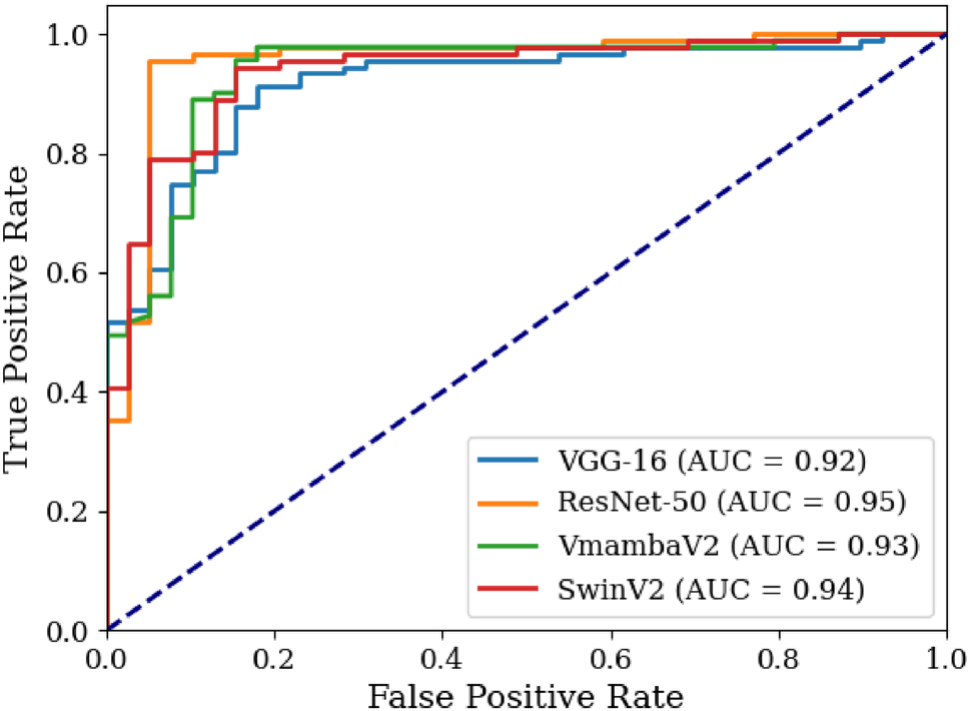
AUROC Curves for Different Backbones.

Classification performance is evaluated using standard metrics, including ACC, PRE, TPR, FPR, F1, and AUC, as summarized in Table 1 and Fig. 3. The results in Table 1 indicate that ResNet-50 outperforms the other models with the highest overall accuracy (0.9462), precision (0.9773), and F1 score (0.9609), along with the lowest false positive rate (0.0513) and the highest AUC (0.9549). VmambaV2 yields the highest TPR (0.9560). SwinV2 achieves the second-best AUC value (0.9377) and the third-best overall classification performance. VGG-16 yields the worst classification performance. ROC curves in Fig. 3 suggest that ResNet-50 is the superior model and SwinV2 is the second-best model for classification.

While tumor classification performance provides some insight, it is not sufficient to fully assess the quality of CAM generation for pseudo label creation. More effectively, we evaluate the segmentation performance of DeepLabV3 trained on pseudo labels generated from these backbone models to gain a comprehensive understanding of their effectiveness in CAM generation. The segmentation performance comparison is compared in Table 2, using Intersection over Union (IoU) and F1 Score as metrics. SwinV2 achieves the highest IoU (0.5426) and F1 score (0.6445), which indicate its superior segmentation performance. The second-best model, ResNet-50, produces significantly lower IoU (0.4607) and F1 score (0.5492). VGG-16 achieves the third-best performance while VmambaV2 ranks as the least effective.

**Table 2:** Segmentation Comparison of Different Backbones.

	IoU$\uparrow$	F1$\uparrow$
SwinV2	**0.5426**	**0.6445**
VmambaV2	0.1510	0.2028
ResNet-50	0.4607	0.5492
VGG-16	0.3502	0.4343

Summarizing the findings from Table 1, Fig. 3, and Table 2, it is evident that classification performance alone does not necessarily correlate with the quality of the generated CAMs or the resulting pseudo labels. For example, although ResNet-50 achieves the highest classification performance, DeepLabV3 trained on its pseudo labels yields only the second-best segmentation results. In contrast, SwinV2, despite lower classification accuracy, enables superior downstream segmentation performance, indicating more reliable CAM localization. Given the focus of this work on accurate segmentation, SwinV2 is therefore selected as the backbone for CAM generation. To further assess generalizability, ResNet-50 is also used in additional experiments.

### Selection of dual-ROI thresholds in DRSeg

This section examines the impact of different threshold values used in the Dual-ROI selection algorithm within the proposed DRSeg framework. As described in section Dual-ROI Selection, the Dual-ROI selection algorithm uses two thresholds, *thres*1 and *thres*2, to identify the most suitable training images for pseudo-segmentation label generation. We set $thres1> thres2$ so that the higher threshold extracts a smaller ROI (*ROI*1, a high-confidence core region), while the lower threshold extracts a larger ROI (*ROI*2, a more inclusive candidate region), as illustrated in Fig. 1. The goal is to select images with two highly overlapping ROIs by using the IoU between them as a quantitative criterion. Since the thresholds directly affect the quality and size of the selected training subset, evaluating their impact is important for achieving robust segmentation performance.

The following two tables summarize the segmentation performance of the proposed DRSeg framework on the BUSI dataset in terms of tumor IoU and F1 score, using SwinV2 as the backbone model. It should be noted that $thres1> thres2$; for example, when $thres1 = 0.8$, we evaluate $thres2 = 0.7$, 0.6, and 0.5. It is unnecessary to include combinations where $thres1 < thres2$ because, in this framework, a pair such as $thres1 = 0.6$ and $thres2 = 0.8$ is equivalent to $thres1 = 0.8$ and $thres2 = 0.6$. This is due to the fact that the overlap between the two extracted ROIs is symmetric and the IoU value remains the same regardless of the order of the thresholds. Therefore, only combinations where $thres1> thres2$ are reported to avoid redundant results.

Table 3 presents the IoU performance of DRSeg across different combinations of *thres*1 and *thres*2. The results show that lower threshold values generally yield smaller IoU scores, whereas intermediate values can lead to significant performance improvements by selecting higher-quality training samples for pseudo-label generation. The highest IoU of 0.5962 is achieved when $thres1 = 0.9$ and $thres2 = 0.7$, indicating that appropriate selection of both thresholds can influence the final segmentation performance.

**Table 3:** IoU performance of the proposed DRSeg framework under different combinations of Dual-ROI thresholds.

thres1	0.5	0.6	0.7	0.8
thres2				
0.6	0.5208	–	–	–
0.7	0.5154	0.5716	–	–
0.8	0.5152	0.5788	0.5618	–
0.9	0.5634	0.5918	**0.5962**	0.4884

Table 4 shows the corresponding F1 score performance under the same threshold combinations. Similar trends are observed, with the F1 score increasing as more suitable training images are selected by the Dual-ROI algorithm. The best F1 score of 0.6898 is obtained with the same combination of $thres1 = 0.9$ and $thres2 = 0.7$, aligning with the IoU results. This consistency further supports the chosen threshold values and highlights the effectiveness of the Dual-ROI algorithm in improving both segmentation precision and recall through careful tuning of both thresholds.

**Table 4:** F1 performance of the proposed DRSeg framework under different combinations of Dual-ROI thresholds.

thres1	0.5	0.6	0.7	0.8
thres2				
0.6	0.6284	–	–	–
0.7	0.6229	0.6720	–	–
0.8	0.6176	0.6744	0.6548	–
0.9	0.6672	0.6820	**0.6898**	0.5842

Summarizing results in both Tables 3 and 4, the combination of $thres1 = 0.9$ and $thres2 = 0.7$ yields the best overall segmentation performance, achieving the highest IoU and F1 scores among all tested combinations. Therefore, this threshold combination is adopted in all subsequent experiments in this study.

### Comparison of Pseudo-label generation methods

Since pseudo-label quality plays an important role in weakly supervised segmentation, we compare several pseudo-label generation methods under the same setting on the BUSI dataset. The goal of this analysis is to isolate the effect of the pseudo-label generator itself, independent of downstream segmentation training.

We evaluate a CAM baseline that directly binarizes CAMs to obtain the larger region of interest (*ROI*2), which serves as a coarse pseudo-label. We further compare SAM-based approaches, including SAM^32^, SAM2^57^, MedSAM^58^, and MedSAM2^59^, where the larger *ROI*2 is used to extract the smallest enclosing bounding box as the spatial prompt. In addition, two classical CAM post-processing methods are included for comparison: DenseCRF refinement and morphological processing. The DenseCRF method treats the normalized CAM as a foreground probability map and refines it using DenseCRF inference before binarization. The morphological baseline applies thresholding followed by morphological closing and retains the largest connected component to form the pseudo segmentation label.

All pseudo labels are generated on the training set consisting of 516 images and are directly compared with the corresponding ground-truth labels to compute the mean IoU and F1 score. This evaluation is intentionally restricted to the training set, as pseudo-label generation is an intermediate step in the training stage and should not involve the test data.

**Table 5:** Comparison of pseudo-label generation methods in terms of mean IoU and F1 score computed against ground-truth labels on the training set of BUSI. Higher values indicate better pseudo-label quality.

Method	IoU $\uparrow$	F1 $\uparrow$
CAM (Baseline)	0.4116	0.5099
SAM	0.4915	0.5882
SAM2	0.4858	0.5899
MedSAM	**0.5153**	**0.6133**
MedSAM2	0.4848	0.5921
DenseCRF	0.3778	0.4873
Morphology	0.3385	0.4351

Table 5 summarizes the quantitative comparison of different pseudo-label generation methods. Overall, SAM-based approaches consistently produce higher-quality pseudo labels than direct CAM binarization and classical post-processing methods. Among the evaluated methods, MedSAM achieves the highest IoU and F1 scores, indicating stronger pseudo-label quality. However, the performance differences among SAM-based variants are relatively modest, suggesting that the quality of the spatial prompt plays a dominant role across different segmentation backbones.

Based on this observation, we adopt SAM as a representative pseudo-label generator in the remaining experiments. While different pseudo-label generators yield varying absolute pseudo-label quality, the proposed image selection strategy operates upstream of pseudo-label generation and is independent of the specific generator employed. Fixing the pseudo-label generator in subsequent experiments allows us to isolate the effect of image selection and avoid confounding factors. Exploring alternative or stronger pseudo-label generators within the DRSeg framework is left for future work.

### Ablation study

This section presents an ablation study to evaluate the contribution of individual components in the proposed DRSeg framework. All experiments are conducted on the BUSI dataset using SwinV2 as the classification backbone, and results are reported as mean ± standard deviation over five random seeds. The quantitative results are summarized in Table 6. The evaluated variants are described as follows:

- **Baseline:** The SwinV2 model is trained for classification and CAM generation. The larger region of interest (*ROI*2) obtained from CAM binarization is directly used as the pseudo segmentation label to train a DeepLabV3 model.
- **Baseline + SAM:** Bounding boxes tightest enclosing *ROI*2 are extracted and used as spatial prompts for the SAM model. The masks generated by SAM are used as pseudo segmentation labels for training DeepLabV3.
- **Baseline + SAM + IRNet:** CAMs generated by SwinV2 are first refined using IRNet. The refined CAMs are then used to obtain *ROI*2, which serves as the spatial prompt for SAM. The remaining steps follow the same procedure as in the “Baseline + SAM” setting.
- **Baseline + SAM + IRNet + Image Selection:** The proposed Dual-ROI selection algorithm is applied to identify a subset of images suitable for pseudo-label generation. Only the selected images are used to generate pseudo labels and train the segmentation model, while the remaining images are excluded at this stage.
- **Baseline + SAM + IRNet + Image Selection + Mean Teacher:** The full DRSeg framework. Both selected and unselected images are used to train DeepLabV3 under the Mean Teacher strategy, where selected images contribute supervised loss via pseudo labels and unselected images contribute through consistency regularization.

**Table 6:** Ablation Study: Performance Impact of Individual Components in the Proposed Framework.

Baseline	SAM	IRNet	Dual-ROI Image Selection	Mean Teacher	IoU$\uparrow$	F1$\uparrow$
$\checkmark$					0.2530 ± 0.0019	0.3738 ± 0.0025
$\checkmark$	$\checkmark$				0.2920 ± 0.0089	0.3878 ± 0.0094
$\checkmark$	$\checkmark$	$\checkmark$			0.5440 ± 0.0066	0.6466 ± 0.0060
$\checkmark$	$\checkmark$	$\checkmark$	$\checkmark$		0.5683 ± 0.0029	0.6707 ± 0.0027
$\checkmark$	$\checkmark$	$\checkmark$	$\checkmark$	$\checkmark$	**0.5935 ± 0.0069**	**0.6927 ± 0.0083**

As shown in Table 6, the baseline yields the lowest segmentation performance, with an IoU of $0.2530 \pm 0.0019$ and an F1 score of $0.3738 \pm 0.0025$, indicating that directly using CAM-derived *ROI*2 as pseudo labels is insufficient for accurate segmentation.

Incorporating SAM improves pseudo-label quality, leading to increased performance (IoU of $0.2920 \pm 0.0089$ and F1 of $0.3878 \pm 0.0094$). A substantial performance gain is observed when IRNet is added for CAM refinement, with IoU and F1 increasing to $0.5440 \pm 0.0066$ and $0.6466 \pm 0.0060$, respectively. This improvement demonstrates that improving CAM quality prior to pseudo-label generation is beneficial for downstream segmentation.

Further improvements are achieved by applying the proposed Dual-ROI image selection algorithm, which increases performance to an IoU of $0.5683 \pm 0.0029$ and F1 of $0.6707 \pm 0.0027$. This result supports our hypothesis of this work: not all training images are equally suitable for pseudo-label generation, and selectively using images with stable and reliable CAM localizations can achieve better segmentation performance.

Finally, the full DRSeg framework incorporating the Mean Teacher strategy achieves the best overall results, with an IoU of $0.5935 \pm 0.0069$ and an F1 score of $0.6927 \pm 0.0083$. This indicates that, while unselected images may not be suitable for pseudo-label generation, they can still provide useful training information through consistency-based regularization. Overall, the ablation study demonstrates that each component of DRSeg contributes meaningfully to the final segmentation performance.

### Comparing segmentation performance with varying subset sizes

This section discusses the segmentation performance of the proposed framework on BUSI under varying subset sizes for pseudo label generation and compare it with fully and semi-supervised learning using the same subsets’ ground truth labels. To assess generalizability, all experiments are conducted using two different classification backbones for CAM generation: SwinV2 and ResNet-50. All results are reported as mean ± standard deviation over five random seeds.

For the proposed weakly supervised learning framework, specifically, a threshold *thres*3 is used to select the most suitable images from the training set for generating pseudo labels, as described in Section Dual-ROI Selection. Increasing the value of *thres*3 results in a smaller subset size for $D_{pseudo}$, as fewer images satisfy the selection criterion. Conversely, a lower *thres*3 value leads to a larger subset size, with more images selected. While a higher *thres*3 value tends to select images with a greater potential for generating high-quality pseudo labels, it also reduces the subset size. On the other hand, a lower *thres*3 value may select more images, but the quality of the generated pseudo segmentation labels could decline. For training a robust and accurate segmentation model, both sufficient training samples and high-quality pseudo labels are important. Therefore, selecting an appropriate value for *thres*3 is the key to balancing the subset size and the quality of pseudo labels.

**Table 7:** SwinV2: Segmentation performance comparison with varying subset sizes on BUSI. Results are reported as mean ± standard deviation over five random seeds. Best results in each column are highlighted in bold.

*thres*3	$D_{\text {pseudo}}$ Size	GT-Subset		Pseudo-Subset		GT-MeanTeacher		Pseudo-MeanTeacher	
		IoU$\uparrow$	F1$\uparrow$	IoU$\uparrow$	F1$\uparrow$	IoU$\uparrow$	F1$\uparrow$	IoU$\uparrow$	F1$\uparrow$
0.1	399	**0.7000 ± 0.0028**	**0.7798 ± 0.0034**	0.5683 ± 0.0029	**0.6707 ± 0.0027**	**0.6837 ± 0.0106**	**0.7714 ± 0.0083**	**0.5935 ± 0.0069**	**0.6927 ± 0.0083**
0.3	307	0.6608 ± 0.0094	0.7368 ± 0.0098	**0.5689 ± 0.0103**	0.6661 ± 0.0119	0.6794 ± 0.0029	0.7617 ± 0.0051	0.5902 ± 0.0111	0.6884 ± 0.0127
0.5	219	0.6481 ± 0.0041	0.7300 ± 0.0043	0.5374 ± 0.0029	0.6319 ± 0.0039	0.6682 ± 0.0157	0.7498 ± 0.0163	0.5526 ± 0.0225	0.6488 ± 0.0310
0.7	152	0.5635 ± 0.0088	0.6381 ± 0.0058	0.5230 ± 0.0153	0.6118 ± 0.0187	0.5870 ± 0.0156	0.6659 ± 0.0197	0.5394 ± 0.0051	0.6365 ± 0.0049
0.9	26	0.4168 ± 0.0108	0.5058 ± 0.0081	0.3627 ± 0.0130	0.4452 ± 0.0235	0.4769 ± 0.0201	0.5593 ± 0.0192	0.3987 ± 0.0050	0.4797 ± 0.0079

Table 7 compares the segmentation performance on BUSI when SwinV2 is used as the backbone. For each value of *thres*3, four training settings are evaluated: **GT-Subset** (fully supervised learning using ground-truth labels of $D_{\text {pseudo}}$), **Pseudo-Subset** (weakly supervised learning using pseudo labels of $D_{\text {pseudo}}$), **GT-MeanTeacher** (semi-supervised learning using ground-truth labels of $D_{\text {pseudo}}$ together with non-pseudo-labeled data), and **Pseudo-MeanTeacher** (the proposed DRSeg framework combining pseudo labels and non-pseudo-labeled data under the Mean Teacher strategy).

Several consistent observations can be made from Table 7. First, using ground-truth labels consistently yields higher segmentation performance than using pseudo labels for the same subset size, as reflected by the superior performance of GT-Subset and GT-MeanTeacher compared to their pseudo-label counterparts. Second, segmentation performance generally decreases as the subset size becomes smaller (i.e., as *thres*3 increases), which is expected due to reduced training data. Third, the Mean Teacher strategy provides substantial and consistent improvements in the weakly supervised setting, as Pseudo-MeanTeacher outperforms Pseudo-Subset across all subset sizes, while its effect in fully supervised learning is comparatively modest and less consistent.

With SwinV2, the proposed weakly supervised DRSeg framework (Pseudo-MeanTeacher) achieves its best performance at $thres3 = 0.1$, corresponding to a subset size of 399 images, with an IoU of $0.5935 \pm 0.0069$ and an F1 score of $0.6927 \pm 0.0083$. At the same subset size, the fully supervised GT-Subset achieves an IoU of $0.7000 \pm 0.0028$ and an F1 score of $0.7798 \pm 0.0034$. Considering the substantial annotation effort required to obtain pixel-wise ground-truth labels and the remaining performance gap of approximately 10% in IoU and 9% in F1, the proposed framework demonstrates a practical balance between annotation cost and segmentation accuracy.

**Table 8:** ResNet-50: Segmentation performance comparison with varying subset sizes on BUSI. Results are reported as mean ± standard deviation over five random seeds. Best results are highlighted in bold.

*thres*3	$D_{pseudo}$ Size	GT-Subset		Pseudo-Subset		GT-MeanTeacher		Pseudo-MeanTeacher	
		IoU$\uparrow$	F1$\uparrow$	IoU$\uparrow$	F1$\uparrow$	IoU$\uparrow$	F1$\uparrow$	IoU$\uparrow$	F1$\uparrow$
0.1	332	**0.7022 ± 0.0016**	**0.7808 ± 0.0032**	0.5264 ± 0.0082	0.6224 ± 0.0119	**0.6932 ± 0.0076**	**0.7768 ± 0.0096**	0.5683 ± 0.0128	0.6680 ± 0.0123
0.3	252	0.6689 ± 0.0106	0.7478 ± 0.0129	0.5244 ± 0.0048	0.6185 ± 0.0071	0.6816 ± 0.0081	0.7603 ± 0.0084	0.5410 ± 0.0083	0.6417 ± 0.0064
0.5	194	0.6288 ± 0.0046	0.7095 ± 0.0037	**0.5492 ± 0.0111**	**0.6443 ± 0.0121**	0.6587 ± 0.0220	0.7380 ± 0.0210	**0.5809 ± 0.0027**	**0.6808 ± 0.0051**
0.7	140	0.5794 ± 0.0290	0.6554 ± 0.0318	0.4887 ± 0.0094	0.5731 ± 0.0095	0.5753 ± 0.0020	0.6468 ± 0.0032	0.5293 ± 0.0148	0.6206 ± 0.0181
0.9	35	0.2810 ± 0.0101	0.3280 ± 0.0106	0.2505 ± 0.0049	0.2892 ± 0.0063	0.3848 ± 0.0409	0.4540 ± 0.0453	0.3434 ± 0.0150	0.4064 ± 0.0204

To further evaluate generalizability across different backbones, Table 8 reports corresponding results on BUSI using ResNet-50 as the backbone. Given that ResNet-50 is a frequently chosen backbone model for various tasks and its strong performance in the backbone selection experiments, as discussed in section Backbone Selection for the Classification Model, it provides a relevant comparison. Overall, the trends observed with SwinV2 remain consistent. In particular, observations regarding the relative effectiveness of ground-truth supervision and the benefit of the Mean Teacher strategy in the weakly supervised setting remain valid. However, the optimal value of *thres*3 differs. For ResNet-50, both Pseudo-Subset and Pseudo-MeanTeacher achieve their best performance at $thres3 = 0.5$, indicating that a moderate subset size provides a better balance between pseudo-label quality and training data quantity for this backbone.

Specifically, Pseudo-MeanTeacher achieves its highest IoU of $0.5809 \pm 0.0027$ and F1 score of $0.6808 \pm 0.0051$ at $thres3 = 0.5$, outperforming training settings with both larger and smaller subsets. In contrast, the fully supervised GT-Subset achieves its best performance at $thres3 = 0.1$, with an IoU of $0.7022 \pm 0.0016$ and an F1 score of $0.7808 \pm 0.0032$. When comparing them at their respective optimal values, the performance gap between fully supervised learning and the proposed weakly supervised framework is approximately 12% in IoU and 10% in F1. When compared under the same subset size ($thres3 = 0.5$), this gap narrows to approximately 5% in IoU and 3% in F1.

Overall, these results demonstrate that the proposed DRSeg framework works across different backbone models while maintaining a consistent trade-off between subset size and pseudo-label quality. Although the optimal value of *thres*3 is backbone-dependent, the selection-driven training strategy consistently enables effective weakly supervised segmentation with reduced annotation effort.

### Performance comparison with Peer methods

In this section, we compare the segmentation performance of the proposed DRSeg framework with representative peer methods on the BUSI and BLU datasets. A fully supervised DeepLabV3 model trained with pixel-level ground-truths is included as an upper-bound reference. Existing weakly supervised segmentation approaches based on image-level supervision can be broadly categorized into CAM-based and multiple instance learning (MIL)-based methods. Accordingly, we select two representative CAM-based methods, AMR^23^ and ReCAM^24^, and two MIL-based methods, SA-MIL^60^ and Swin-MIL^45^, for comparison.

It should be noted that the two CAM-based methods were originally designed for natural images and the two MIL-based methods were originally designed for biopsy images. To the best of our knowledge, no existing method has been specifically designed for weakly supervised BUS image segmentation with publicly available code. Therefore, these four methods are chosen as the most relevant available benchmarks for comparison. In addition, we evaluate four variants of the proposed DRSeg framework: ResNet and SwinV2 backbones trained with and without the Dual-ROI image selection strategy. The variants incorporating Dual-ROI image selection represent the full versions of DRSeg.

**Table 9:** Segmentation Performance Comparison with Peer Methods. The best weakly supervised methods are in bold.

Methods	BUSI		BLU	
	IoU$\uparrow$	F1$\uparrow$	IoU$\uparrow$	F1$\uparrow$
Fully Supervised DeepLabV3	0.7134 ± 0.0045	0.7959 ± 0.0035	0.7232 ± 0.0111	0.8229 ± 0.0122
ReCAM	0.1062 ± 0.0113	0.1423 ± 0.0146	0.0480 ± 0.0297	0.0661 ± 0.0398
AMR	0.1843 ± 0.0026	0.2595 ± 0.0024	0.1153 ± 0.0221	0.1777 ± 0.0302
SA-MIL	0.0873 ± 0.0036	0.1487 ± 0.0053	0.0630 ± 0.0092	0.1146 ± 0.0151
Swin-MIL	0.0991 ± 0.0012	0.1658 ± 0.0020	0.0638 ± 0.0099	0.1144 ± 0.0173
ResNet w/o Dual-ROI Selection	0.4684 ± 0.0084	0.5626 ± 0.0071	0.3403 ± 0.0490	0.4416 ± 0.0507
SwinV2 w/o Dual-ROI Selection	0.5440 ± 0.0066	0.6466 ± 0.0060	0.4973 ± 0.0575	0.5970 ± 0.0657
ResNet w/Dual-ROI Selection (proposed)	0.5837 ± 0.0092	0.6841 ± 0.0102	**0.5179 ± 0.0318**	0.6000 ± 0.0284
SwinV2 w/Dual-ROI Selection (proposed)	**0.5935 ± 0.0069**	**0.6927 ± 0.0083**	0.5118 ± 0.0480	**0.6060 ± 0.0574**

Table 9 presents a segmentation performance comparison between DRSeg and peer methods on the BUSI and BLU datasets over five random seeds. As expected, the fully supervised DeepLabV3 model achieves the highest performance across both datasets, serving as a performance upper bound. All weakly supervised methods perform substantially worse than the fully supervised baseline, highlighting the inherent difficulty of BUS image segmentation under image-level supervision.

Among the peer weakly supervised methods, CAM-based approaches (AMR and ReCAM) generally outperform MIL-based approaches (SA-MIL and Swin-MIL), although their overall performance remains limited, with IoU values below 0.20 on BUSI and below 0.15 on BLU. This performance gap may be attributed to the fact that these methods were not specifically designed for BUS images, which are characterized by low contrast, speckle noise, and ambiguous tumor boundaries.

The proposed DRSeg framework demonstrates clear performance advantages over all peer weakly supervised methods across both datasets. For a fixed backbone, incorporating the Dual-ROI image selection strategy consistently improves the segmentation performance. On BUSI, ResNet-based DRSeg improves from $0.4684 \pm 0.0084$ to $0.5837 \pm 0.0092$ in IoU, while SwinV2-based DRSeg improves from $0.5440 \pm 0.0066$ to $0.5935 \pm 0.0069$ in IoU. Similar trends are also observed for F1 scores. These results indicate that selectively filtering training images based on CAM stability leads to more reliable pseudo labels and improved downstream segmentation.

On the BLU dataset, DRSeg also achieves the best performance among all weakly supervised methods. ResNet-based DRSeg attains the highest IoU ($0.5179 \pm 0.0318$), while SwinV2-based DRSeg achieves the highest F1 score ($0.6060 \pm 0.0574$). Compared with their counterparts without Dual-ROI selection, ResNet- and SwinV2-based DRSeg show consistent performance improvements, although the magnitude of improvement varies across backbones and datasets. These results suggest that backbone choice can influence performance across datasets, while the proposed Dual-ROI image selection algorithm provides consistent benefits.

**Table 10:** Statistical significance on BUSI. Differences are reported as $\Delta$IoU (Proposed − Peer) with 95% confidence intervals. Paired tests are conducted across five matched random seeds with Holm correction.

Methods	$\Delta$IoU	95% CI	Sig.
ReCAM	0.4872	(0.4662, 0.5082)	$$^{***}$$
AMR	0.4092	(0.4035, 0.4148)	$$^{***}$$
SA-MIL	0.5061	(0.4958, 0.5165)	$$^{***}$$
Swin-MIL	0.4944	(0.4852, 0.5036)	$$^{***}$$
ResNet w/o Dual-ROI Selection	0.1251	(0.1104, 0.1397)	$$^{***}$$
SwinV2 w/o Dual-ROI Selection	0.0495	(0.0426, 0.0564)	$$^{***}$$

**Table 11:** Statistical significance on BLU. Differences are reported as $$\Delta$$ (Proposed − Peer) with 95% confidence intervals. Paired two-sided tests are conducted across five matched random seeds with Holm correction. $$^{***}p<0.001$$, $$^{**}p<0.01$$, $$^{*}p<0.05$$, n.s.: not significant.

Methods	$\Delta$IoU	95% CI	Sig.
ReCAM	0.4638	(0.4040, 0.5235)	$$^{***}$$
AMR	0.3965	(0.3406, 0.4524)	$$^{***}$$
SA-MIL	0.4488	(0.3878, 0.5098)	$$^{***}$$
Swin-MIL	0.4480	(0.3869, 0.5091)	$$^{***}$$
ResNet w/o Dual-ROI Selection	0.1715	(0.0752, 0.2678)	$^{*}$
SwinV2 w/o Dual-ROI Selection	0.0145	(−0.0472, 0.0762)	n.s.

To further assess the robustness of these improvements, we conduct statistical significance analysis using paired tests across five matched random seeds. Tables 10 and 11 report the IoU differences between the proposed DRSeg (SwinV2 with Dual-ROI selection) and each peer method, together with 95% confidence intervals and Holm-corrected significance results. On BUSI, DRSeg achieves statistically significant improvements over all peer methods as well as over its variants without Dual-ROI selection ($p<0.001$). On BLU, improvements over CAM-based and MIL-based methods remain statistically significant, whereas the improvement over SwinV2 without Dual-ROI selection is not statistically significant, reflecting the smaller performance gap observed on this dataset.

**Fig. 4:**
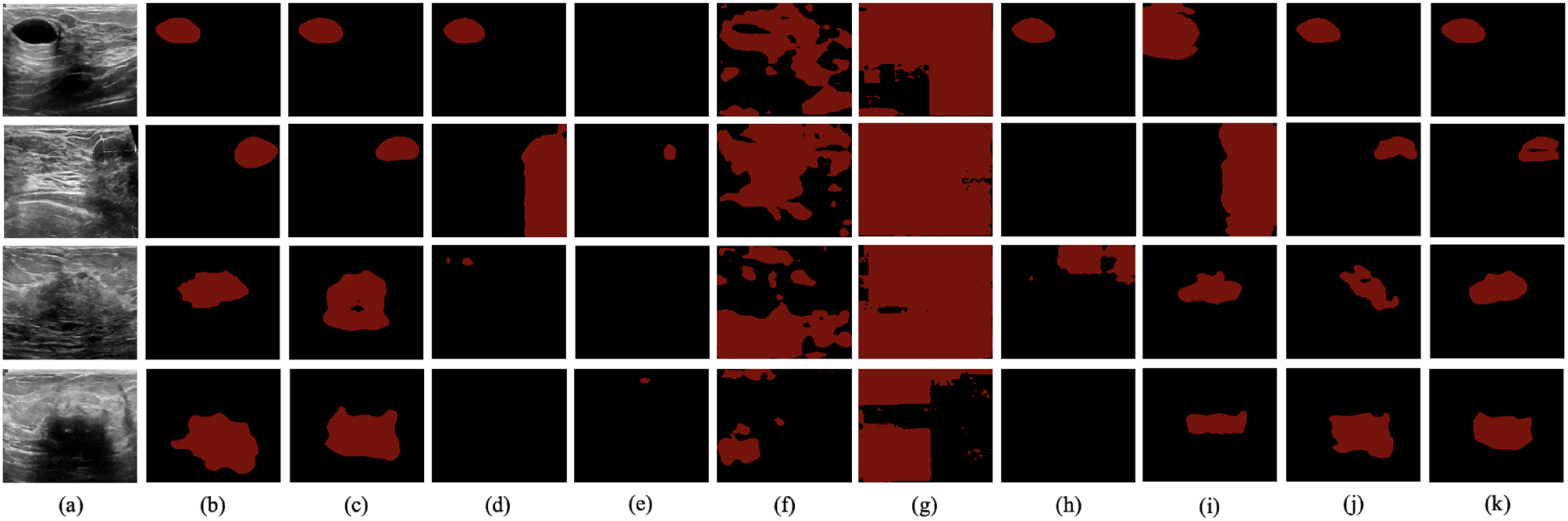
(**a**) Original BUS images, (**b**) Ground truth, and (**c**–**k**) segmentation results generated by different methods: (**c**) Fully Supervised DeepLabV3, (**d**) ReCAM, (**e**) AMR, (**f**) SA-MIL, (**g**) Swin-MIL, (**h**) ResNet without Image Selection, (**i**) SwinV2 without Image Selection, (**j**) ResNet with Image Selection (proposed), and (**k**) SwinV2 with Image Selection (proposed).

Figure 4 presents a qualitative visual comparison of segmentation performance for all methods listed in Table 9. It displays four representative BUS images from the BUSI dataset along with their corresponding ground truths and segmentation results generated by each method. The first BUS image contains a benign tumor with clear and smooth boundary. The second BUS image contains a benign tumor in the top-right corner, exhibiting a relatively low contrast with the surrounding tissue. The third and fourth images contain malignant tumors with irregular shapes and blurry boundaries.

The fully supervised DeepLabV3 model (column k) produces relatively good segmentation results for all BUS images, except for the third row. Among the weakly supervised methods, the two CAM-based (ReCAM, AMR) and two MIL-based (SA-MIL, Swin-MIL) approaches (columns d–g) produce poor segmentation results. ResNet without Dual-ROI selection and SwinV2 without Dual-ROI selection (columns h and i) are able to catch the tumor location in most cases but fail to accurately segment the tumors. The proposed DRSeg framework with ResNet and SwinV2 as backbones (columns j and k) achieves the most accurate segmentation among all weakly supervised methods, though its performance remains lower than the fully supervised approach. The qualitative results are consistent with the quantitative analysis and further demonstrate that explicitly selecting images with stable CAM localizations improves segmentation reliability under weak supervision.

Overall, the results in this subsection demonstrate that DRSeg improves weakly supervised BUS image segmentation by explicitly incorporating the Dual-ROI image selection algorithm, a selection-driven training strategy. By filtering unreliable pseudo labels prior to segmentation training, DRSeg achieves consistent improvements across datasets and backbones, highlighting its effectiveness and practical potential for reducing annotation cost in BUS image analysis.

### Cross-dataset generalization evaluation

To further examine the generalization capability of the proposed DRSeg framework under domain shift, we conduct cross-dataset segmentation experiments between the BUSI and BLU datasets. We aim to evaluate DRSeg’s ability to transfer learned representations across datasets with different data distributions and imaging characteristics.

Based on the results discussed in Performance Comparison with Peer Methods, SwinV2 is selected for DRSeg trained on BUSI, while ResNet-50 is selected for DRSeg trained on BLU, as these backbones achieve the best overall performance on their respective datasets. Specifically, DRSeg with a SwinV2 backbone is trained on BUSI and tested on BLU, and DRSeg with a ResNet-50 backbone is trained on BLU and tested on BUSI. In both cases, the full DRSeg framework with all components is applied during training using the same experimental settings.

**Table 12:** Cross-dataset generalization performance of DRSeg under different backbone training configurations.

Training $\rightarrow$ Testing	IoU$\uparrow$	F1$\uparrow$
BUSI $\rightarrow$ BLU (SwinV2 backbone)	0.4698	0.5634
BLU $\rightarrow$ BUSI (ResNet backbone)	0.3463	0.4421

As shown in Table 12, DRSeg trained on BUSI and tested on BLU achieves an IoU of 0.4698 and an F1 score of 0.5634. Conversely, DRSeg trained on BLU and tested on BUSI achieves an IoU of 0.3463 and an F1 score of 0.4421. Although these results are lower than the corresponding in-dataset evaluations, this performance drop is expected due to the substantial domain gap between the two datasets.

These results indicate that DRSeg shows measurable transfer performance under domain shift, though lower than in-domain evaluation. This evaluation is intended as an exploratory analysis of generalization behavior rather than a statistically rigorous comparison. Accordingly, results are reported from a single training run without variance estimation.

## Discussion

This work presents DRSeg, a weakly supervised training framework for BUS image segmentation that addresses a key limitation of CAM-based approaches: the large variability in pseudo-label quality across training images. Rather than introducing a new segmentation architecture, DRSeg augments a standard CAM-based pipeline with an explicit image selection stage that identifies samples capable of producing reliable pseudo labels. Experimental results consistently show that not all images are equally suitable for pseudo-label generation, and that selectively training on images with stable CAM localizations leads to substantial improvements in segmentation performance.

The major contribution of DRSeg is the proposed Dual-ROI image selection algorithm, which quantifies CAM stability across threshold perturbations using spatial overlap. This simple yet effectively criterion filters out images with unstable activation maps, thereby reducing pseudo-label noise before segmentation training. Across ablation studies, subset size analyses, and comparisons with peer methods, Dual-ROI selection consistently improves segmentation accuracy, demonstrating that selection-driven training is a practical and effective strategy in weakly supervised settings.

The integration of the Mean Teacher strategy further enhances DRSeg by allowing both pseudo-labeled and non-pseudo-labeled images to contribute during training. Results indicate that the Mean Teacher strategy provides the largest benefits when combined with reliable pseudo labels, reinforcing the importance of improving pseudo-label quality prior to segmentation optimization. In contrast, its effect is smaller and less consistent in fully supervised learning, suggesting its primary benefit is to stabilize weakly supervised learning.

Experimental analyses also suggest that optimal configurations, including backbone choice and selection thresholds, can be dataset-dependent. For example, the SwinV2 backbone yields stronger CAM-based segmentation on BUSI, while the ResNet backbone performs better on BLU. DRSeg naturally accommodates such variability, as its selection mechanism and thresholds can be adjusted to balance subset size and pseudo-label quality for different datasets and backbones.

Statistical significance analysis further supports these findings. DRSeg achieves consistent and statistically significant improvements over existing weakly supervised segmentation methods across two datasets, BUSI and BLU. Image selection helps the most when pseudo labels are noisy. If the backbone is already strong and pseudo labels are relatively stable, the improvement becomes smaller and more dataset-dependent. While the present study is intentionally scoped to weakly supervised breast ultrasound segmentation using publicly available datasets, extending the proposed framework to broader datasets, imaging modalities, and evaluation settings represents an important direction for future research.

Additional experiments comparing different pseudo-label generation methods indicate that stronger generators, such as MedSAM, can produce higher-quality pseudo labels. However, evaluating all downstream settings with multiple pseudo-label generators would make it difficult to isolate the effect of the proposed Dual-ROI image selection algorithm. Therefore, a fixed pseudo-label generator (SAM) is used in most experiments, while DRSeg is designed to remain compatible with alternative generators in future work.

Finally, cross-dataset experiments indicate that DRSeg retains measurable segmentation capability under domain shift, even without target-domain supervision. The observed performance degradation across datasets is likely influenced by differences in image contrast, noise level, and lesion appearance, which can reduce CAM stability and pseudo-label quality. Since DRSeg relies on stable localization for image selection, such degradation naturally affects the downstream segmentation performance. These cross-dataset results are exploratory, and isolating individual factors under controlled backbone and threshold settings remains future work.

Beyond breast ultrasound imaging, the core ideas of DRSeg, including explicit image selection, backbone-aware optimization, and effective use of weak supervision, are applicable to other medical imaging tasks where pixel-wise annotations are costly. Future work will extend DRSeg to additional datasets and imaging domains and explore adaptive thresholding and stronger pseudo-label generators to further improve segmentation reliability while reducing annotation effort.

## Conclusion

This work proposes DRSeg, a weakly supervised training framework for breast ultrasound image segmentation. The core contribution is a Dual-ROI image selection algorithm that identifies training images with stable CAM localizations, enabling the generation of more reliable pseudo labels prior to segmentation training. Experimental results demonstrate that not all images are equally suitable for producing reliable pseudo labels, highlighting the importance of an explicit image selection strategy for improving pseudo-label quality and, consequently, segmentation performance. The proposed Dual-ROI image selection algorithm is model-independent and can be readily integrated into existing CAM-based weakly supervised segmentation methods. In addition, experimental results show that both pseudo-labeled and non-pseudo-labeled images contribute to effective segmentation training. Accordingly, DRSeg employs the Mean Teacher strategy to leverage both pseudo-labeled and non-pseudo-labeled images during training. Overall, the proposed DRSeg framework demonstrates the potential to reduce the time and labor required for pixel-wise annotation while achieving competitive segmentation performance across two public datasets and two backbone models. These results suggest that selection-driven training provides a practical and effective direction for weakly supervised breast ultrasound image segmentation.

## Data Availability

The data used in this research are publicly available. The code will be made available from the corresponding author on reasonable request.
